# Prevalence of dementia, heart disease and stroke in community-dwelling adults in Canada, 2016–2021: opportunities for joint prevention

**DOI:** 10.1186/s13690-023-01171-7

**Published:** 2023-08-24

**Authors:** Sarah S. Singh, Shiran Zhong, Kem A. Rogers, Vladimir C. Hachinski, Stephanie J. Frisbee

**Affiliations:** 1 Robarts Research Institute, University of Western Ontario, 100 Perth Dr, London, ON N6A 5K8 Canada; 2 Department of Geography, University of Western Ontario, London, ON Canada; 3 Department of Anatomy & Cell Biology, Schulich School of Medicine & Dentistry, University of Western Ontario, London, ON Canada; 4 Department of Clinical Neurological Sciences, and Epidemiology and Biostatistics, Schulich School of Medicine & Dentistry, University of Western Ontario, London, ON Canada; 5 Department of Pathology & Laboratory Medicine, and Epidemiology & Biostatistics, Schulich School of Medicine & Dentistry, University of Western Ontario, London, ON Canada

**Keywords:** Dementia, Heart disease, Stroke, Vascular disease, Risk factors, Prevalence, Trends, Prevention

## Abstract

**Introduction:**

This aim of this study is to provide updated estimates on the prevalence of dementia, heart disease, and stroke in Canadian communities. Targeting all three conditions together, at the community level, may be key to disease prevention and health aging in the Canadian population.

**Methods:**

Using nationwide health survey data, we calculated the age-standardized prevalence of self-reported dementia, heart disease and stroke in adults aged 18 years and over residing in Canadian communities from 2016 to 2021. Poisson regression models were used to detect statistically significant changes in the prevalence of all three conditions from 2016 to 2021.

**Results:**

Less than 1% (~ 175,000 individuals) of adults residing in Canadian communities reported dementia, 5% (~ 1.5 million individuals) reported heart disease, and more than 1% (~ 370,000 individuals) reported stroke annually from 2016 to 2021. Overall, the age-standardized prevalence for stroke decreased minimally from 2016 to 2021 (p = 0.0004). Although the age-standardized prevalence of heart disease and dementia decreased from 2016 to 2018, subsequent increases in prevalence from 2018 to 2021 led to a lack of overall statistically significant changes from 2016 to 2021 (p = 0.10 for heart disease and p = 0.37 for dementia).

**Conclusion:**

Recent increases in the prevalence of dementia, heart disease and stroke in Canadian communities threaten to reverse any gains in vascular disease prevention over the past six years. Findings reveal the urgent need for intensified prevention efforts that are community-based with a focus on joint reduction in the shared risk factors contributing to all three diseases.

**Supplementary Information:**

The online version contains supplementary material available at 10.1186/s13690-023-01171-7.

**Table:** 

**Text box 1. Contributions to the literature**
• Vascular diseases, including dementia, heart disease and stroke, are some of the leading causes of hospitalization and death in Canada and worldwide.
• Despite previous reports of declining rates of vascular disease over the past decade, findings of this study reveal that the prevalence of dementia, heart disease and stroke have remained persistently high, with no significant improvement in the past six years (2016–2021).
• This study adds to existing literature by providing an urgent update on the prevalence of leading vascular diseases in Canadian communities, where disease prevention can have the greatest impact.

## Introduction

In 2020, approximately 20% of deaths in Canada were attributed to three diseases, dementia, heart disease and stroke, with the majority of deaths and hospitalizations occurring in those aged 65 years and over [1]. Given the recent increase in global life expectancy, meeting the health care needs of the older population in Canada over the next ten years is expected to add $93 billion to health care costs [2]. As the Canadian population ages, enabling individuals to remain healthy in their homes and communities for as long as possible can prove beneficial for policy makers, health care providers, families and seniors themselves. However, up to 60% of those aged 65 years and over, currently living at home in Canada, report having one chronic disease and 37% report having at least two chronic diseases [3]. Many of these chronic conditions, such as hypertension, can be managed through pharmaceutical intervention and lifestyle modifications thereby preventing progression to debilitating conditions, such as stroke. Consequently, there is an urgent need for greater efforts to support healthy aging in place, in part, through chronic disease prevention and health promotion in Canada.

Numerous studies have demonstrated a strong link between vascular diseases, such as dementia, heart disease and stroke. A 2018 systematic review and meta-analysis of over one million individuals in North America and Europe revealed a 27% increased risk of dementia for those with vs. without a history of coronary heart disease (HR 1.27; 95% CI [1.07–1.50]) [4]. Furthermore, studies have shown the protective effect of a healthy heart on brain disease. Research on a subset of adults aged 58 years and over from the UK Rotterdam Study, showed that higher diastolic function in the heart was associated with a reduced risk of both stroke (HR 0.82; 95% CI [0.69–0.98] and dementia (HR 0.82; 95% CI [0.70–0.96]) [5]. Additionally, researchers estimated that approximately 40% of dementia cases were attributable to preventable comorbid diseases including heart disease and stroke [6]. Given that the prevalence of dementia, heart disease and stroke increases with age, examining the concurrence of multiple vascular diseases may be a key step towards meeting the healthcare needs of an aging population. This concept was recently endorsed in a call to action by the World Health Organization (WHO), and many other international organizations, emphasizing that the greatest reduction in dementia risk could arise from early interventions to prevent stroke and heart disease [7, 8].

National estimates from 2021 indicate that more than 90% of Canadian seniors dwell in their adult homes and remain embedded in their communities [9, 10]. Furthermore, 61% of seniors with dementia live at home, as opposed to long term care institutions [3]. The WHO defines healthy aging as “the process of developing and maintaining the functional ability that enables well-being in older age”. According to WHO, this functional ability is determined by the “intrinsic capacity of the individual (i.e., the combination of all the individual’s physical and mental – including psychosocial – capacities), the environments he or she inhabits (understood in the broadest sense and including physical, social and policy environments), and the interaction between these”. Therefore, concept of aging in place should occur along the life course and provides a unique opportunity to implement primary and secondary prevention of vascular diseases at both the individual and community levels [11]. Results from randomized trials have successfully demonstrated that pharmacological interventions and lifestyle modifications have led to reductions in systolic blood pressure and vascular disease mortality rates at the individual level [12, 13]. Furthermore, studies have confirmed that short-term community-based programmes have effectively reduced cardio-metabolic risk through lifestyle modification at the community level [14]. Understanding the burden of vascular diseases in community-dwelling adults, who have not yet been institutionalized, is the first step in guiding public health policy, implementing programs and allocating resources towards healthy aging in place.

A recent study on dementia in community-dwelling Canadian adults revealed an overall increase in the prevalence of dementia from 1994 to 2013, followed by a decrease in 2014 [15]. Additionally, the study reports a greater increase in dementia prevalence for males than females, with a switch noted in later years [15]. Another study demonstrated a decline in the age- and sex-adjusted prevalence of both heart disease and stroke from 2005 to 2016 in community-dwelling Canadians [16]. Similar results were noted for institutionalized adults, with hospitalization rates for heart disease and stroke decreasing from 2007 to 2016, and increasing for cognitive impairment during the same time period [17]. Few studies have examined the prevalence of dementia, heart disease and stroke concurrently and no Canadian studies extend beyond the year 2016. The primary aim of this study was to provide an update on the prevalence of dementia, stroke and heart disease in community-dwelling Canadians from 2016 to 2021. The secondary aim of the study was to examine sex differences and changes in the prevalence of all three diseases over the 6-year study period. Findings from this study will provide an updated outlook on the burden of vascular diseases in Canadian adults residing at home, and encourage an early, joint prevention plan for tackling dementia, heart disease and stroke at the community level to support healthy aging in place.

## Methods

This study uses nationally representative data from the Canadian Community Health Survey (2016–2021) to describe the prevalence of self-reported dementia, heart disease and stroke in adults aged 18 years and over, dwelling in Canadian communities and not institutionalized into long term care.

### Study Sample and Data source

The study sample included all Canadians aged 18 years and over from the annual Canadian Community Health Survey (CCHS) files for cycles 2016–2021. The CCHS is a national cross-sectional survey that utilizes multistage sampling to facilitate national estimates on self-reported health data. The CCHS data includes those who reside in private dwellings in 10 provinces and 3 territories. The CCHS data excludes individuals living on Indian Reserves and Crown lands, clientele of institutions, the full-time members of the Canadian Forces, and residents of some remote areas. Ineligible participants included children and youth aged less than 18 years and those with missing data on dementia, heart disease and stroke. Given the medical nature of dementia, eligible participants included those with proxy responses which represented less than 10% of overall responses.

### Study outcomes

The three study outcomes were dementia, heart disease and stroke. The following questions were asked to study participants: “Do you have Alzheimer’s Disease or any other dementia?”, “Have you ever been diagnosed with heart disease?”, and “Do you suffer from the effects of a stroke?”, All questions were asked under the premise of conditions diagnosed by a health professional and that were expected to last or have already lasted 6 months or more. For the purposes of this study, participants who responded “yes” to these questions were classified as having dementia, heart disease and stroke respectively.

### Study covariates

The study covariates were age and sex. Age in years were classified according to 5-year age groups from 18 to 25 years, 25–30 years, until the age of 80 years and over. Sex was classified as assigned sex at birth, male or female. For descriptive purposes, education was classified as less than secondary, secondary or post-secondary education. Total household income was classified as $0–19,999, $20,000–59,999, $60,000–99,000, at or above $100,000. Race was classified as White, South Asian, Chinese, Black, Filipino, Latin American, Arab, Southeast Asian, West Asian, Korean, Japanese, Visible minority not otherwise listed and Multiple visible minorities.

### Statistical analysis

#### Study population

The weighted proportion and weighted frequency of the population reporting dementia, heart disease and stroke were calculated for each of the six survey cycles (2016–2021). The study population has also been described based on categories of study covariates.

#### Concurrence of Dementia, Heart Disease and Stroke

To examine the concurrence of dementia, stroke and heart disease, we calculated crude numbers across the six survey cycles (2016–2021), overall and for males and females, for the following subgroups: [1] individuals reporting heart disease and stroke, no dementia, [2] individuals reporting dementia and stroke, no heart disease, [3] individuals reporting dementia and heart disease, no stroke, [4] individuals reporting all three conditions; dementia, stroke and heart disease.

#### Prevalence of Dementia, Heart Disease and Stroke

For each survey cycle, crude prevalence rates were calculated for dementia, heart disease and stroke separately. To account for changes in the age structure of the Canadian population over the study period, age-standardized prevalence rates (per 1000 individuals) for dementia, heart disease and stroke were calculated for each survey cycle from 2016 to 2021. Estimates were derived using the direct method and the 2016 Canadian standard population calculated from Census data. Differences in age-standardized rates from 2016 to 2021 were then tested using Poisson regression models that included survey cycle as a continuous variable (p-value for significance < 0.05).

#### Prevalence of Dementia, Heart Disease and Stroke in Males and Females

Analyses for age-standardized prevalence rates were repeated as above and stratified by sex to produce age-standardized prevalence rates (per 1000 individuals) for dementia, heart disease and stroke in males and females separately. Sex differences in age-standardized rates across the five survey cycles were then tested using Poisson regression models that included an interaction term for sex and survey cycle (p-value for significance ≤ 0.05).

Statistical analyses were performed using SAS version 9.4 (SAS Institute, Cary, NC, USA). All descriptive estimates and confidence intervals were derived using the sampling weights and bootstrap weights provided by the Canadian Community Health Survey (2016–2021) to account for the complex, multistage sampling design and obtain precise estimates of variation.

## Results

### Study population

Descriptive characteristics of the study sample for each survey cycle (2016–2021) are shown in Table 1. The majority of the adult population was aged 40–59 years, with postsecondary education, and White Non-Hispanic race/ethnicity for each survey cycle. Less than 1.0% (~ 131,000–196,000 individuals) of households reported dementia, 4.6-5.2% (~ 1.4–1.6 million individuals) reported heart disease, and 1.2-1.4% (~ 360,000–398,000 individuals) reported stroke from 2016 to 2021.

**Table 1:** Characteristics of the study population by year from 2016–2021, Canadian Community Health Survey

Characteristics		2016 (n = 28,433,497)	2017 (n = 28,750,507)	2018 (n = 29,143,122)	2019 (n = 29,580,349)	2020 (n = 29,861,825)	2021 (n = 30,130,680)
**Study covariates**							
Sex	Male	49.2(13,982,631)	49.1(14,143,360)	49.2(14,342,784)	49.2(14,566,434)	49.3(14,730,741)	49.3(14,863,101)
	Female	50.8(14,450,866)	50.8(14,607,147)	50.8(14,800,337)	50.8(15,013,916)	50.7(15,131,084)	50.7(15,267,580)
Age (years)	18–24	10.6(3,032,736)	10.4(3,023,819)	10.4(3,051,075)	10.6(3,124,033)	10.0(2,974,476)	10.0(3,015,742)
	25–39	25.9(7,377,661)	26.3(7,577,991)	26.4(7,700,309)	25.8(7,660,382)	26.6(7,945,372)	26.0(7,834,973)
	40–59	35.3(10,003,809)	34.0(9,779,418)	33.5(9,783,677)	33.5(9,945,130)	32.3(9,672,041)	32.1(9,670,275)
	60–79	24.2(6,881,727)	24.8(7,110,469)	25.1(7,337,848)	25.3(7,488,763)	26.4(7,873,095)	27.4(8,149,363)
	80 & over	4.0(1,137,566)	4.3(1,258,811)	4.4(1,270,213)	4.9(1,360,940)	4.6(1,396,842)	4.8(1,460,329)
Education	Less than secondary	12.3(3,502,136)	11.3(3,254,160)	11.1(3,235,131)	10.7(3,155,008)	9.4(2,805,195)	9.0(2,722,930)
	Secondary	24.3(6,911,214)	24.8(7,119,759)	23.6(68,887,981)	23.5(6,963,398)	22.8(6,794,006)	22.5(6,789,713)
	Post Secondary	61.6(17,516,982)	62.1(17,862,430)	63.5(18,513,160)	64.2(19,003,874)	66.7(19,911,781)	67.6(20,373,311)
	Not stated	1.8(503,166)	1.8(514,158)	1.7(506,850)	1.5(458,069)	1.2(350,844)	0.8(244,725)
Total household income ($)	≤ 19,999	7.3(2,085,959)	6.5(1,873,850)	6.2(1,814,736)	4.5(1,356,824)	3.6(1,103,556)	4.0(1,225,122)
	20,000–59,999	28.3(8,053,212)	27.6(7,928,244)	27.7(8,086,953)	27.3(8,059,647)	25.0(9,431,206)	22.3(6,753,773)
	60,000–99,000	25.1(5,324,323)	24.6(7,072,485)	23.7(6,916,846)	24.4(7,193,298)	24.4(7,270,556)	23.2(6,990,084)
	≥ 100,000	39.2(11,143,201)	40.9(11,763,865)	42.3(12,324,585)	43.5(12,891,899)	46.6(13,907,214)	50.2(15,128,249)
Race and ethnicity	White Non-Hispanic	72.4(20,599,954)	73.0(20,993,125)	71.9(20,952,458)	71.1(21,040,806)	76.9(22,957,601)	74.5(22,441,757)
	South Asian	4.2(1,206,146)	4.3(1,247,638)	5.2(1,513,333)	5.0(1,479,352)	5.4(1,601,574)	5.9(1,784,742)
	Chinese	4.1(1,165,713)	4.0(1,138,562)	4.2(1,228,845)	4.3(1,278,590)	4.4(1,321,830)	4.8(1,449,321)
	Black	2.4(673,691)	2.5(723,473)	2.6(756,425)	2.9(858,442)	3.0(894,214)	3.2(960,525)
	Filipino	2.0(558,729)	2.2(645,979)	2.1(622,352)	1.9(567,908)	2.1(622,710)	2.4(728,253)
	Latin American	1.3(365,697)	1.5(438,781)	1.2(362,570)	1.6(472,267)	1.2(368,267)	1.5(441,410)
	Arab	1.3(355,550)	1.2(341,889)	1.3(381,322)	1.3(388,445)	1.7(509,738)	1.8(557,723)
	Southeast Asian	1.1(325,453)	1.2(337,640)	0.9(273,913)	1.0(308,517)	1.2(350,437)	1.3(404,688)
	West Asian	0.7(199,559)	0.5(153,391)	0.7(205,686)	0.6(187,912)	0.6(174,723)	0.7(203,690)
	Korean	0.4(113,720)	0.5(138,511)	0.5(136,018)	0.3(86,025)	0.4(108,411)	0.4(121,054)
	Japanese	0.2(67,263)	0.3(74,063)	0.3(77,700)	0.2(62,863)	0.3(86,658)	0.3(96,300)
	Other Visible minority	1.4(386,739)	1.4(396,620)	1.7(486,981)	1.9(556,312)	0.2(73,837)	0.2(70,566)
	Multiple visible minorities	2.0(561,396)	2.2(620,229)	1.9(571,383)	1.9(570,773)	0.5(162,548)	0.8(246,510)
	Not stated	3.0(861,025)	1.7(494,331)	1.9(556,047)	2.5(734,108)	2.1(629,278)	2.1(624,141)
	Valid skip	3.5(993,273)	3.5(1,006,273)	3.4(1,018,088)	3.3(988,028)	-	-
**Study outcomes**							
Alzheimer’s and other dementias	Reported yes	0.6(176,461)	0.5(153,371)	0.5(136,049)	0.6(175,805)	0.4(131,721)	0.7(197,446)
Heart disease	Reported yes	5.1(1,438,398)	5.0(1,437,606)	4.6(1,348,708)	4.8(1,427,249)	5.2(1,541,138)	5.2(1,571,299)
Stroke	Reported yes	1.3(362,452)	1.4(388,822)	1.3(374,671)	1.3(398,512)	1.3(383,887)	1.2(366,493)

*Weighted percentages (%) and weighted frequencies (n) were derived using the CCHS sampling weights to adjust for the complex survey design of the CCHS.

### Concurrence of Dementia, Heart Disease and Stroke

**Figs. 1:**
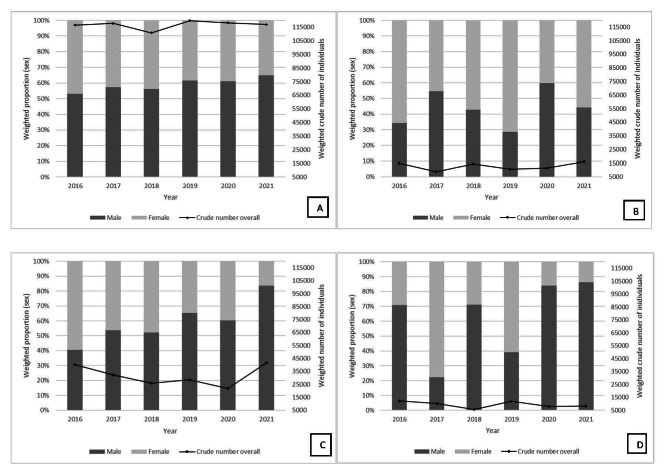
**A-D**. Crude Number of Individuals with Dementia, Heart Disease and Stroke in Males and Females from 2016–2021: **A**. Heart Disease and Stroke, **B**. Dementia and Stroke, **C**. Dementia and Heart Disease, **D**. Dementia, Heart Disease and Stroke, Canadian Community Health Survey. *Weighted frequencies (n) were derived using the CCHS sampling weights to adjust for the complex survey design of the CCHS. A – Number of individuals with Heart Disease and Stroke. B – Number of individuals with Dementia and Stroke. C – Number of individuals with Dementia and Heart Disease. D – Number of individuals with Dementia, Stroke and Heart Disease

Figure 1 A-D demonstrate the crude number of individuals reporting heart disease and stroke, dementia and stroke, dementia and heart disease, and all three conditions. Overall, the number of individuals reporting heart disease and stroke was the highest concurrence of conditions from 2016 to 2021. Additionally, more males than females reported concurrent conditions, including all three conditions, over time.

For heart disease and stroke, the crude number increased from 116,527 to 116,976 from 2016 to 2021, with a peak of 119,758 in 2019. For dementia and stroke, the crude number increased from 15,142 to 16,148 from 2016 to 2021, with a peak of 16,148 in 2021. For dementia and heart disease, the crude number increased from 40,092 to 41,621 from 2016 to 2021, with a peak of 41,621 in 2021. For dementia, heart disease and stroke, the crude number decreased from 12,429 to 8203 from 2016 to 2021, with a peak of 12,429 in 2016.

### Prevalence of Dementia, Heart Disease and Stroke

**Fig. 2:**
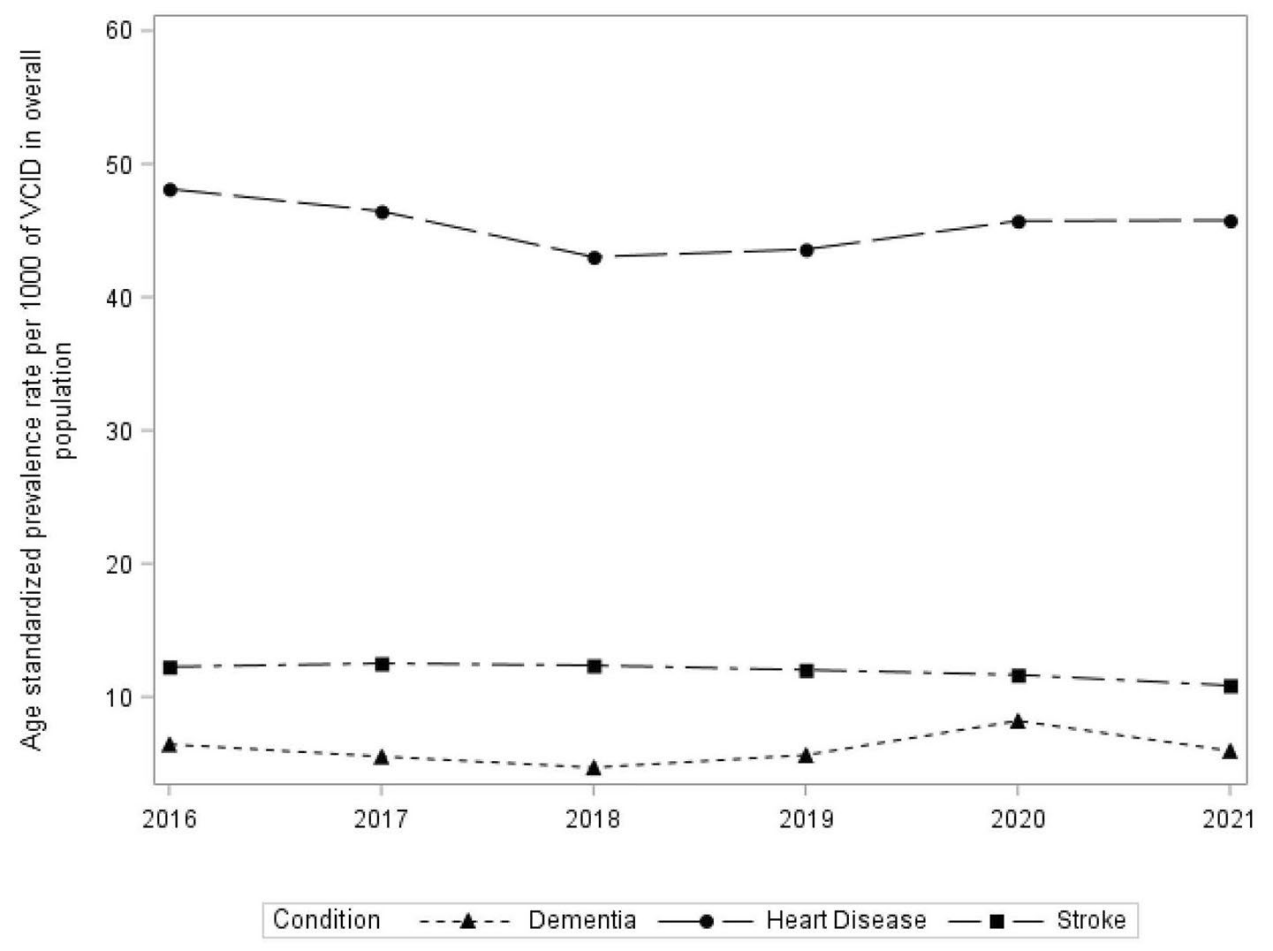
Age-standardized Prevalence Rates of Dementia, Stroke and Heart Disease from 2016 to 2021 in Canadian adults aged 18 years and older, Canadian Community Health Survey. *Weighted frequencies (n) were derived using the CCHS sampling weights to adjust for the complex survey design of the CCHS.

Figure 2 demonstrates age-standardized prevalence rates of dementia, heart disease and stroke over the six-year study period (crude and age-standardized prevalence rate point estimates were reported in Supplementary Table). Prevalence rates were highest for heart disease, followed by stroke and lowest for dementia in all survey cycles. Overall, the prevalence stroke decreased steadily. However, the prevalence of heart disease and dementia decreased from 2016 to 2018 and increased from 2018 to 2021.

Dementia rates peaked in 2020 (8.20 (95% CI,8.17–8.22)), while stroke (12.51(95% CI,12.47–12.55)) and heart disease rates (48.12(95% CI,48.04–48.20)) peaked in 2017 and 2016 respectively. Overall, prevalence rates did not statistically differ from 2016 to 2021 for heart disease (p-value = 0.42) and dementia (p-value = 0.27). Age standardized prevalence rates decreased minimally from 2016 to 2021 for stroke only (p-value = 0.0004).

### Prevalence of Dementia, Heart Disease and Stroke in Males and Females

**Fig. 3:**
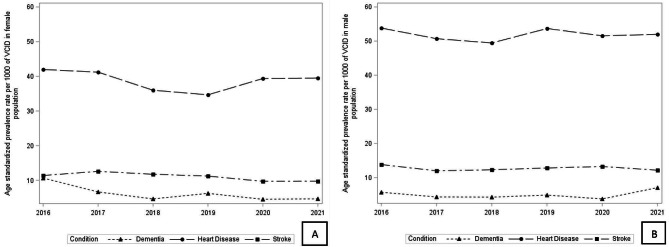
**A-B** Age-standardized Prevalence Rates of Dementia, Heart Disease and Stroke in Males and Females from 2016–2021 in Canadian adults 18 years and older, in females and males respectively, Canadian Community Health Survey. *Weighted frequencies (n) were derived using the CCHS sampling weights to adjust for the complex survey design of the CCHS. ┼Figure 1 A: Females, Fig. 1B: Males

Sex-specific age standardized prevalence rates of dementia, stroke and heart disease over the six-year study period are shown in Fig. 3A-B (age-standardized prevalence rate point estimates for males and females were reported in Supplementary Table). Overall, dementia rates were higher for females, whereas stroke and heart disease rates were higher for males. Prevalence rates for dementia increased in males (p-value = 0.009) and decreased in females (p-value = 0.0009) from 2016 to 2021. Prevalence rates for stroke decreased significantly in females only (p-value = 0.04) from 2016 to 2021. Heart disease prevalence rates did not change significantly over time in males or females.

In females, the prevalence of dementia (10.64 (95% CI,10.59–10.71)) and heart disease (41.97 (95% CI,41.87–42.08)) were highest in 2016, while stroke rates were highest in 2017 (12.58 (95% CI,12.53–12.64)). In males, the prevalence of dementia (7.07 (95% CI,7.04–7.11) was highest in 2021, while stroke (13.82 (95% CI,13.76–13.88)) and heart disease (53.82 (95% CI,53.70-53.93)) rates were highest in 2016.

## Discussion

In a nationwide study of community-dwelling Canadian adults, we found that the prevalence of self-reported dementia, heart disease and stroke decreased in earlier years (2016 to 2018), however, this pattern was reversed in later years (2018 to 2021). Despite declining trends reported in previous decades, overall, in this study, there were no significant changes noted in the prevalence of dementia and heart disease from 2016 to 2021, and only a minimal decrease noted for stroke. Sex-based differences in trends were noted for dementia only, where females reported a decrease in dementia prevalence while males reported an increase in dementia prevalence from 2016 to 2021. Additionally, the number of individuals with at least two concurrent conditions increased, while the number of individuals with all three conditions decreased, from 2016 to 2021. Results of the study confirm little to no progress in the prevention of vascular disease in Canadian communities over the past six years.

Our findings are consistent with research demonstrating an increase in the prevalence of dementia in recent years in Canadian communities and institutions. A previous study by Chambers-Richards et al. reported on the trends in dementia in non-institutionalized individuals from 1994 to 2014 in Canada [18]. Results of that study indicated an overall increase in dementia prevalence over the study period, with greater increases noted for females as compared to males in later years. Another study using administrative data from Ontario, found an increase in the prevalence of dementia within the community from 2010 to 2015, also with greater increases noted for females [15]. Similarly, other studies using health administrative data from Ontario and Saskatchewan, capturing institutionalized individuals, revealed findings to support an increase in dementia prevalence prior to 2016 [15, 19]. While our study did not find any significant changes in dementia prevalence since 2016, there was a trend towards increased prevalence from 2018 to 2021. Conversely, our study adds that females experienced a decrease in the prevalence of dementia, a pattern that was reversed in males in later years.

Few other studies have investigated the prevalence of dementia alongside that of heart disease and stroke in the same study. Using Global Burden of Disease (GBD) data, Morovatdar et al. found that the prevalence of dementia, heart disease and stroke together increased from 1990 to 2017 globally [20]. Similar to our study, authors found that stroke and heart disease were more prevalent in men, while dementia was more prevalent in women. Additionally, authors confirm that while trends in prevalence rates for all three diseases flattened in earlier years, significant increases in trends were noted after 2013. Our study adds to these findings beyond 2013, demonstrating increases in the number of individuals with two concurrent diseases particularly in the last year of the study. Our conclusions concur with those of the GBD study, confirming the ongoing need to address the growing burden of vascular diseases in Canada through prevention targeting dementia, heart disease and stroke simultaneously. Results may be explained, in part, by the aging population which supports a call for greater disease prevention efforts that promote aging in place within communities.

Our study found that the number of individuals with two concurrent conditions increased over time. Additionally, there were significant, and even alternating, sex-based differences in the number of individuals with all three conditions over time. Such findings may be, at least in part, explained by an exponentially increased risk of mortality associated vascular comorbidities [21, 22]. Results of our study do not confirm, but support the premise of heart disease and stroke as risk factors for dementia. In 2017, Singh-Manoux et al. demonstrated that atrial fibrillation was associated with accelerated cognitive decline. However, this association did not remain significant after accounting for stroke and coronary heart disease [23]. Other recent studies demonstrate that the most significant risk factor for cognitive decline after a stroke was experiencing a second stroke [24]. The pathophysiology underlying vascular changes in the heart and degenerative changes in the brain is highly complex and may occur jointly or even cumulatively [7]. It is, therefore, crucial for clinicians, researchers and public health authorities to employ interventions towards individuals with high vascular burden for the prevention of detrimental clinical consequences.

Study findings also revealed that while dementia was more prevalent in women, stroke and heart disease were more prevalent in men. Similar results for dementia have been noted worldwide, with women outnumbering men almost 2 to 1 in some regions [25]. Studies have attributed this finding to the longer life expectancy in women, however, newer factors including gender discrimination and disability are now being considered as important targets for action in reducing dementia burden [26, 27]. Furthermore, we found that when considering those with all three diseases, the burden was more equitably distributed between men and women in alternating years. The risk factors for vascular disease and dementia, such as lifestyle and behavioral factors, are the effectively the same in men and women, however. disease and risk factor prevalence vary significantly between the sexes. Therefore, therapies for risk factor reduction may require a more sex-specific approach. For example, in 2004 the American Heart Association released women-specific evidence-based guidelines on the prevention of cardiovascular disease [28]. However, a 2011 update of those guidelines indicated that very few intervention studies recorded sex-specific differences in efficacy, making progress difficult to measure [29].

Finally, early intervention is key in addressing vascular diseases simultaneously. Recent interventional studies, such as the ASPIS trial (Austrian Polyintervention Study to Prevent Cognitive Decline after Ischemic Stroke) and the PODCAST trial (Prevention of Decline in Cognition after Stroke Trial), did not demonstrate improved cognitive ability following the implementation of lifestyle changes, including lowering blood pressure, after stroke [30, 31]. However, A 2017 ARIC study (Atherosclerosis Risk in Communities) found a significant association between midlife vascular risk factors and elevated amyloid deposition in the brain in later years [32]. Results of these studies emphasize the considerable role for primordial and primary prevention, that is, early risk factor control preceding signs of clinical vascular disease. Therefore, healthy aging must occur within a life-course framework, where risk factor control and lifestyle modification implemented in early life can slow or halt progression to chronic disease and functional decline in later life.

Over the past few decades, there has been growing support at the global and national levels for the concept of aging in place as an ideal path for healthy aging. Today in Canada, there is consensus that caring for the elderly population in an institutional setting, such as long-term care, is not optimal given the decreased quality of life and increased costs attached [33]. However, aging in place requires careful strategy and planning which involves the provision of appropriate resources such as healthcare, housing, transportation, community support [34]. Results from this study have shown persistently high rates of vascular disease in Canadian communities and points to the need for greater health promotion and disease prevention services to facilitate aging in place [35]. Provinces have initiated local interventions aimed at addressing modifiable factors, such as physical activity and social support in the high-risk elderly population [36]. Further work is still needed to fund nationwide initiatives for promoting healthy lifestyle, support research in healthy aging and healthy communities, build partnerships to strengthen community resources available to seniors and establish national guidelines for healthy aging in local communities [37].

### Strengths and Limitations

A major strength of the study is the use of nationally representative data consisting of participants across the entire of Canada. Results from this study provide a true and updated representation of the current vascular burden within Canadian communities. A limitation of this study was the exclusion of the institutionalized population from the study sample. The institutionalized population consists of some of Canada’s most aged individuals, with an average age of 83 years old [38]. Census statistics indicate that, in 2016, up to 160,000 adults lived-in long-term care in Canada and this number is projected to increase to 199,000 by 2035 [39, 40]. Therefore, as the Canadian population ages, more individuals may be living in long term care rather than aging in their communities which may have impacted the prevalence rates presented in this study.

Additionally, recent report by Statistics Canada indicated that up to 45% of individuals aged 45 years and older living in long term care facilities report dementia, with a higher proportion of older women due to longer life expectancies [41]. Therefore, the estimation of dementia prevalence in the community is likely an underestimation of the entire national dementia prevalence. However, authors believe that primary disease prevention is most impactful at the community level where interventions may prevent progression to hospitalization and mortality [42]. Therefore, it is important to estimate community prevalence of these conditions.

Furthermore, the use of annual period prevalence to examine trends as opposed to a longitudinal follow-up over time still provides a cross sectional view on trends in dementia, stroke and heart disease at the population level. Therefore, any temporal effect of heart disease and stroke as causal factors for development of dementia could not be assessed and was beyond the scope of this study. Additionally, the self-reported nature of the conditions may lead to reporting bias and an underestimation of the true population prevalence of these conditions. Finally, our study uses a wide definition for dementia – Alzheimer’s and any other dementia – with no specific reference to vascular dementia. However, our intention was to capture a national picture of overall dementia burden given the emergence of mixed dementia [43].

## Conclusion

There has been no significant improvement in the prevalence of dementia, heart disease or stroke in the past six years in Canadian communities. However, there has been a growing number of individuals reporting concurrent vascular diseases. Additionally, the prevalence of vascular disease in males in recent years has exceeded that of females. Future interventions to address the high prevalence of vascular disease may benefit from a sex-based approach that jointly addresses dementia, heart disease or stroke at the community level over a sustained period. Such an approach would incorporate screening and early detection of shared risk factors, with a focus on how these factors influence the prevalence of vascular disease in males and females differently.

### Electronic supplementary material

Below is the link to the electronic supplementary material.

**Supplementary Table**: Crude and Age standardized prevalence rates (per 1000 individuals) of dementia, heart disease and stroke in the total sample and males and females independently.

## Data Availability

All analyses were conducted within the Research Data Center at the University of Western Ontario, part of the Canadian Research Data Centre Network, the Canada wide platform that provides secured access to microdata for research and training purposes. Access to and use of these microdata are governed by the Statistics Act, the Privacy Act, and the Access to Information Act. Researchers are only permitted to access anonymized data; thus, all projects are considered exempt from ethics review. Finally, while academic researchers are able to access data within the Canadian Research Data Centre Network/Research Data Center sites free of charge, full compliance with all regulations and requirements for access, use, and reporting of the data, as specified by the Canadian Research Data Centre Network and Statistics Canada, is required. Information about accessing Statistics Canada microdata is available here: https://www.statcan.gc.ca/en/microdata/data-centres/access. Information about accessing data through the Research Data Center at the University of Western Ontario is available here: https://rdc.uwo.ca/data_access/index.html. Additional data requests may be directed to the corresponding author Sarah Singh at ssing452@uwo.ca.
