# Supplementary material for: Prevalence of dementia, heart disease and stroke in community-dwelling adults in Canada, 2016–2021: opportunities for joint prevention
Source: Arch Public Health. 2023 Aug 24;81:158. doi: 10.1186/s13690-023-01171-7 (PMC10464200; doi:10.1186/s13690-023-01171-7)
Supplement: Supplementary file 1 — Supplementary Table: Crude and Age standardized prevalence rates (per 1000 individuals) of dementia, heart disease and stroke in the total sample and males and females independently. [file 13690_2023_1171_MOESM1_ESM.docx]

**Supplementary Table** Crude and Age standardized prevalence rates (per 1000 individuals) of dementia, heart disease and stroke in the total sample and males and females independently.

|  | 2016 | 2017 | 2018 | 2019 | 2020 | 2021 |
| --- | --- | --- | --- | --- | --- | --- |
| All | | | | | | |
| Dementia | 5.75, 6.44 (6.41-6.47) | 4.95, 5.52 (5.49-5.54) | 4.33, 4.69 (4.66-4.72) | 5.52, 5.63 (5.60-5.65) | 6.71, 8.20 (8.17-8.22) | 5.58, 5.95 (5.92-5.98) |
| Stroke | 11.99, 12.26 (12.22-12.30) | 12.57, 12.51 (12.47-12.55) | 12.21, 12.36 (12.32-12.40) | 12.53, 12.02 (12.02-12.10) | 8.89, 11.65 (11.61-11.69) | 10.46, 10.86 (10.83-10.90) |
| Heart Disease | 47.33, 48.12 (48.04-48.20) | 46.80, 46.45 (46.37-46.52) | 43.63, 43.02 (42.95-43.10) | 45.13, 43.59 (43.52-43.66) | 35.69, 45.72 (45.64-45.80) | 44.79,45.77 (45.70-45.84) |
| Male | | | | | | |
| Dementia | 5.21, 5.71 (5.67-5.75) | 2.61, 4.34 (4.30-4.37) | 4.23, 4.28 (4.25-4.32) | 5.60, 4.89 (4.86-4.93) | 4.02, 3.76 (3.73-3.79) | 7.67, 7.07 (7.04-7.11) |
| Stroke | 13.52, 13.82 (13.76-13.88) | 7.62, 12.00 (11.95-12.05) | 12.82, 12.30 (12.24-12.35) | 14.51, 12.83 (12.77-12.88) | 14.31, 13.26 (13.21-13.31) | 13.05, 12.18 (12.12-12.23) |
| Heart Disease | 54.07, 53.82 (53.70-53.93) | 31.33, 50.71 (50.60-50.82) | 51.58, 49.46 (39.34-49.57) | 59.45, 53.69 (53.56-53.82) | 56.08, 51.54 (51.43-51.64) | 57.24, 51.98 (51-87-52.09) |
| Female | | | | | | |
| Dementia | 8.21, 10.64 (10.59-10.71) | 5.43, 6.65 (6.60-6.69) | 4.43, 4.64 (4.60-4.67) | 5.84, 6.23 (6.20-6.28) | 4.15, 4.52 (4.48-4.56) | 4.50, 4.66 (4.63-4.70) |
| Stroke | 11.07, 11.39 (11.34-11.45) | 12.14, 12.58 (12.53-12.64) | 11.10. 11.75 (11.70-11.81) | 11.57, 11.22 (11.17-11.27) | 9.70, 9.71 (9.66-9.76) | 9.81, 9.73 (9.68-9.78) |
| Heart Disease | 40.76, 41.97 (41.87-42.08) | 40.25, 41.20 (41.10-41.31) | 35.78, 35.99 (35.90-36.09) | 35.21, 34.66 (34.56-34.75) | 40.32, 39.37 (39.27-39.46) | 40.77, 39.49 (39.39-39.59) |

*Estimates are presented as crude rate, age standardized rate (95% CI)
